# Evaluation of Therapeutic Opioids in Skin‐Derived Matrices (Sweat and Sebum) of Neonatal and Pediatric Patients and Their Role in Opioid Incorporation Into Hair

**DOI:** 10.1002/dta.70034

**Published:** 2026-02-26

**Authors:** Max Polke, Florian Zapf, Julia T. Scherer, Clarissa D. Voegel, Tanja Restin, Marc W. Schmid, Thomas Kraemer, Tina M. Binz

**Affiliations:** ^1^ Center for Forensic Hair Analytics, Zurich Institute of Forensic Medicine University of Zurich Zurich Switzerland; ^2^ Department for Pediatric Intensive Care Medicine and Neonatology University Children's Hospital Zurich Zurich Switzerland; ^3^ Institute of Physiology and Newborn Research University of Zurich Zurich Switzerland; ^4^ MWSchmid GmbH Glarus Switzerland; ^5^ Department of Forensic Pharmacology and Toxicology, Zurich Institute of Forensic Medicine University of Zurich Zurich Switzerland

**Keywords:** children, fentanyl, hair analysis, LC–MS/MS, opioids

## Abstract

Hair analysis is a valuable tool in forensic toxicology to assess opioid exposure in both adult and pediatric populations. However, interpretation of analysis results is challenged by the scarcity of reliable reference data on opioids in hair and by the lack of knowledge on involved hair incorporation pathways, e.g., via skin‐derived matrices such as sweat and sebum. In order to address these limitations, we conducted a clinical study in cooperation with the University Children's Hospital Zurich, where we obtained and analyzed hair and skin swab samples of 150 pediatric intensive‐care patients (median age: 53 days). These patients had a treatment history with opioids, including fentanyl, sufentanil, remifentanil, alfentanil, morphine, methadone, and hydromorphone. Most of the substances, as well as selected metabolites, were repeatedly detected in both sample types, exhibiting comparable concentration trends and metabolite‐to‐parent drug ratios. For fentanyl and morphine, a positive correlation was observed between the administered opioid doses and the analyte concentrations in both the hair and skin swab samples. In addition, a correlation was found for fentanyl between concentrations in hair and skin swab samples (*r* = 0.356, *p* = 0.0007), indicating a contribution of skin‐derived matrices to opioid incorporation into the hair of children and neonates. The comprehensive and clinical nature of this study provides unique value for the generated concentration data and associated findings, providing a potential reference source for future interpretation of hair analysis results.

## Graphical abstract

Concentration ranges, dose–concentration relationships, and metabolite‐to‐parent drug ratios of therapeutic opioids were evaluated in hair and skin swab samples of 150 pediatric patients. Skin‐derived matrices such as sweat or sebum were shown to contribute to analyte incorporation into hair in a systematic manner. These findings address a critical knowledge gap in forensic hair analysis and provide an important basis for the future interpretation of hair analysis results in children and neonates.

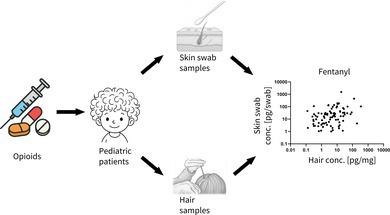

## 1 Introduction

Hair analysis is widely used in forensic toxicology and can provide crucial information in the investigation of drug‐facilitated crimes, child custody cases, and prevalence studies of emerging drugs of abuse [1, 2]. The key advantage of this keratinized matrix over conventional ones such as blood or urine is that drugs and potential metabolites, once embedded into the keratin structure of the hair, remain chemically stable and protected from rapid (metabolic) degradation. This enables substances to be detected over extended time periods (several months) [3].

These advantages are contrasted by the fact that interpreting analysis results for this matrix is comparatively challenging. This is mainly due to the complexities underlying substance incorporation into hair. In this context, three different incorporation pathways can be distinguished [2]. The first one involves active or passive diffusion of substances from the bloodstream into the dermal papilla of the growing hair. Hair grows approximately 10 mm per month [2]. As a result, substances incorporated through this mechanism require approximately 7–10 days to reach the scalp surface. The second pathway entails the diffusion of substances, dissolved in skin‐derived matrices such as sweat and sebum on the scalp, into the hair shaft. In contrast to the incorporation via the bloodstream, this mechanism can already be relevant hours after a substance intake [2]. Thirdly, substances may also incorporate through external contamination of the hair by vapors or powders, etc. In most cases, an interplay of all three mechanisms takes place [4, 5]. The contribution of these pathways and the extent of incorporation is highly individual for substances and depends on several parameters such as membrane permeability, lipophilicity, and melanin affinity [6]. Incorporation is further influenced by the physical characteristics of the individual or population under investigation [7]. For instance, children's hair is comparatively more porous in its structure, which favors radial diffusion of substances from sweat and sebum or external sources of contamination [7]. Notably, this can distort the relationship between hair concentrations and the extent of an individual's substance intake or exposure [7].

Substance‐specific literature on these complexities and comparative data, such as commonly observed concentration ranges, dose–hair concentration relationships, and metabolite‐to‐parent drug ratios, can essentially support the interpretation of hair analysis results. Despite the high prevalence and considerable forensic relevance of opioids, such literature is scarce for this substance class. This holds especially true for vulnerable populations such as children and neonates who are unfortunately regularly harmed by intentional and unintentional intake and exposure to opioids, as documented by various reports [7, 8, 9, 10, 11, 12].

In order to address this limitation, a clinical study in cooperation with the University Children's Hospital Zurich has been initiated. This study enrolled children and neonates that recently underwent medication with opioids (fentanyl, sufentanil, remifentanil, alfentanil, morphine, hydromorphone, and methadone) as part of their clinical treatment. From these patients, we aimed to collect and analyze both hair and skin swab samples and obtain concentration data of the administered opioids and selected metabolites of fentanyl (norfentanyl, β‐hydroxyfentanyl, and 4‐anilino‐N‐phenethylpiperidine [4‐ANPP]), sufentanil (norsufentanil), and morphine (hydromorphone) in hair and skin‐derived matrices such as sweat and sebum. Previously, 118 hair samples from this cohort were analyzed, and initial results were obtained. These preliminary findings were published and discussed together with the developed and validated hair analysis method by Polke et al. [1]. The present study focuses on the development and validation of a liquid chromatography–tandem mass spectrometry (LC–MS/MS)‐based skin swab analysis method and the subsequent analysis of the authentic skin swab samples. Based on the complete dataset, comprising the analysis results of 150 hair and skin swab samples, we aim to evaluate potential similarities, differences, and correlations between the analyte concentrations of the two sample types. This comparative approach allows for the investigation of incorporation mechanisms, in particular the role of skin‐derived matrices and their contribution to opioid incorporation into hair. Moreover, we aim to investigate the potential influence of various patient‐ and treatment‐related covariates on the analyte concentrations in the matrices, which has either never been investigated or has only been disclosed to a limited extent for this population. Specifically, these covariates include patients' age, gender, hair color, and special life‐supporting treatments (organ support therapies).

## 2 Material and Methods

### 2.1 Study Design and Cohort Inclusion

During this noninterventional prospective study, hair and skin swab samples were obtained from 150 children and neonates from the Pediatric and Neonatal Intensive Care Unit (P/NICU) of the University Children's Hospital Zurich. Details on the inclusion criteria of the study, the hair sampling procedure and analysis method, as well as information on the collected patient data, can be obtained from Polke et al. [1]. In brief, study inclusion was possible up to 13 years of age and previously administered medication had to include either fentanyl, sufentanil, remifentanil, alfentanil, or more traditional opioids such as morphine. Detailed patient and medication‐related data in a pseudonymized form was collected, namely, date of birth, weight, gender, hair color, application of organ support therapies, the type and total cumulative amount (μg per kilogram body weight [μg/kgBW]) of the specific opioids administered, duration of medication, duration since the last opioid administration, and potential intrauterine exposure of mothers to opioids during birth or C‐section. The study received ethical approval by the Swiss Ethics Board (approval number: 2022‐01693/amendment approval date: 09.01.2024) and was registered at ClinicalTrial.gov (Identifier NCT05740657, released on 13.02.2023).

### 2.2 Reference Substances and Chemicals

Standard solutions in methanol or acetonitrile (1 mg/mL) of 4‐ANPP, acetylcodeine, 6‐monoacetylmorphine, alfentanil, dihydrocodeine, fentanyl, hydrocodone, hydromorphone, methadone, morphine, and pethidine were obtained from Cerilliant (Round Rock, USA). Reference standards of codeine, naloxone, norfentanyl, oxycodone, oxymorphone, and tramadol, as well as deuterated standards (0.1 mg/mL) of fentanyl‐d_5_, methadone‐d_9_, and morphine‐d_3_, were purchased from Lipomed (Arlesheim, Switzerland). Methanolic solutions (1 mg/mL) of norsufentanil, remifentanil, remifentanil acid, and sufentanil and 0.1mg/mL solutions of the deuterated standards norfentanyl‐d_5_ and norsufentanil‐d_3_ were obtained from Cayman Chemical (Ann Arbor, USA). β‐Hydroxyfentanyl (1 mg/mL) was purchased from LGC (Wesel, Germany). LC–MS‐grade methanol (Chromasolv) used for the preparation of working solutions was obtained from Sigma‐Aldrich (Buchs SG, Switzerland). LC–MS‐grade acetonitrile was sourced from ACROS ORGANICS (Fisher Scientific AG, Switzerland), LC–MS‐grade water (Chromasolv) from Sigma‐Aldrich, and ammonium formate and formic acid were purchased from Merck (Darmstadt, Germany) for use in LC–MS/MS mobile phases.

### 2.3 Preparation of Calibrators and Quality Control (QC) Samples

Based on the LLOQ values defined in the sensitivity experiments described below, the analytes were assigned to one out of six groups (see Table S1). Stock solutions containing nondeuterated analytes were prepared at 200 ng/mL for groups 1–5 and 2000 ng/mL for group 6. Further, a methanolic internal standard (IS) stock solution containing fentanyl‐d_5_, norfentanyl‐d_5_, norsufentanil‐d_3_, methadone‐d_9_, and morphine‐d_3_ each at a concentration of 40 pg/μL was prepared. IS and stock solutions were stored at −20°C in amber vials until further use. QCs and calibrators (C1–C8) were freshly prepared before each run by spiking blank skin swabs (medical cotton swabs [Forensic Swab L, SARSTED, Germany]) with dilutions of stock solutions and 50‐μL IS solution. The resulting calibration ranges and QC levels (QC_low_, QC_med_, and QC_high_) are listed in Table S2. Blank skin swabs were obtained from drug‐abstinent volunteers who had given oral consent at the Center for Forensic Hair Analytics, Zurich, Switzerland.

### 2.4 Hair and Skin Swab Sampling and Sample Preparation

Hair sampling followed the established protocol of the Center for Forensic Hair Analytics [13]. For sample preparation and extraction, a so‐called one‐pot approach was employed, where pulverization and extraction are performed in the same vessel (Eppendorf tube) to minimize the risk of external contamination and sample loss [14]. Details on the applied procedures can be obtained from our previous publication on the preliminary analysis results of the hair samples by Polke et al. [1]. The preservation method for skin swab samples is based on the procedure described by Jarmusch et al. [15]. Particular care was taken to ensure that the sampling procedure was both comfortable and minimally invasive for the participants. Prior to sampling, the swabs were dipped in an ethanol/water mixture (1:1). Sampling was performed by gently rubbing the skin surface (forehead, behind one ear, neck, and between fingers) in a circular motion for approximately 10 s with moderate and painless pressure. The swabs were then sealed in the original sampling container and stored at −20°C until further processing. Prior to sample extraction, the tip of each cotton swab was cut and placed into a 2‐mL Eppendorf tube. To each tube, 1.5 mL of methanol (MeOH) and 50 μL of IS solution (40 pg/μL) were added. The tubes were then shaken in an Eppendorf Thermomixer F2.0 (Eppendorf, Geneva, Switzerland) at 1500 rpm for 15 min at 23°C. After extraction, the supernatants were transferred to glass tubes. A second extraction was performed by adding an additional 1.5 mL of fresh MeOH to the swabs, followed by the same shaking procedure. The resulting supernatants from both extraction steps were combined and evaporated to dryness under nitrogen flow (2 bar) at 35°C for 45 min. The dried residues were reconstituted in 150 μL of MeOH and 350 μL of reconstitution solution. The samples were then centrifuged at 9000 rpm for 10 min using 0.45‐μm centrifugal filter tubes (modified nylon, VWR, USA). Finally, the filtrates were transferred to LC–MS/MS screw neck vials for analysis. This sampling approach entails variability in the amount of analytes obtained (refer to the limitations chapter).

### 2.5 Instrumentation

For the analysis of the skin swab samples, the same chromatographic conditions and largely similar MS parameters as for the hair sample analysis were applied [1] and are summarized as follows: A Prominence UFLC system (Shimadzu, Kyoto, Japan) equipped with a Kinetex F5 column (100 mm × 2.1 mm, 100 Å, 2.6 μm, Phenomenex) coupled with an ultrahigh performance liquid chromatography (UHPLC) SecurityGuard ULTRA Cartridge F5 (2.1‐mm ID) was employed. The column and the autosampler were maintained at 40°C and 15°C, respectively. The binary mobile phase consisted of A: water with 1‐mM ammonium formate and 0.1% formic acid, and B: acetonitrile with 1‐mM ammonium formate and 0.1% formic acid. The sample injection volume was 5 μL, and the subsequent gradient elution was at a flow rate of 0.6 mL/min using the following time program: 0–1.5 min maintaining eluent B at 3%; 1.5–9 min increasing to 60% eluent B; 9–10 min increasing to 95% eluent B; 10–11 min maintaining eluent B at 95%; 11.01 min returning to starting conditions (3% eluent B) and maintaining the gradient until the end of the run at 13 min.

For analyte detection, a QTRAP 7500 from Sciex (Darmstadt, Germany) was operated in multiple reaction monitoring (MRM) mode, utilizing positive electrospray ionization at 1500 V. Nitrogen was used as the curtain gas (fixed at 42 psi), and the source (OptiFlow Pro) was maintained at a temperature of 500°C. For identification and quantification, two individually optimized MRM transitions were used for each analyte, applying a detection window of ±15 s around the respective elution time. The retention times and MS parameters can be obtained from Table S3.

### 2.6 Method Validation

The validation experiments performed in this work were based on the guidelines of the Society of Toxicological and Forensic Chemistry (GTFCh) [16, 17]. Validation took place in terms of selectivity, specificity (including matrix effects), sensitivity (limit of detection [LOD] and lower limit of quantification [LLOQ]), linearity, accuracy, precision (intraday and interday), and recovery (extraction efficiency). Furthermore, stability of the analytes was evaluated with regard to freezing and thawing of the skin swab samples.

### 2.7 Statistical Analysis

Statistical analyses were conducted using Prism 10 (GraphPad Software, CA, USA). Prior to correlation analysis, corresponding data were assessed for normality using the Shapiro–Wilk test. As the data did not follow a normal distribution in all cases, nonparametric Spearman's rank correlation coefficients were calculated. *p*‐values > 0.05 were considered as not statistically significant (ns); *p* < 0.05 as significant; *p* < 0.01 as very significant; *p* < 0.001 and *p* < 0.0001 as extremely significant.

## 3 Results and Discussion

### 3.1 Validation of the Analytical Method for Skin Swab Samples

The developed LC‐method was capable of adequately separating all 22 analytes. Analysis of six different drug‐free human skin swab samples showed no interference signals at the corresponding retention times of the analytes. Calibration measurements in the low concentration range demonstrated good sensitivity of the method for most analytes, with signals exceeding a signal‐to‐noise (S/N) ratio of 3:1 (determining the LOD), at analyte concentrations ranging from 0.2 to 4 pg/swab. In terms of the LLOQ, ratios greater than 10:1 (S/N) were evaluated for concentrations from 1.0 to 10 pg/swab. Methadone represented the only exception with LOD and LLOQ analyte concentrations of 40 and 60 pg/swab, respectively. Details on sensitivity values and calibration ranges can be obtained from Table S1. The method demonstrated substantially higher sensitivity compared to similar validated analytical skin swab approaches described in the literature, which report LODs exceeding 70 pg/swab and LLOQs above 210 pg/swab [18, 19].

Calibration models showed good linearity (*R* ^2^ > 0.98) within the calibration ranges. Given the complexity of the matrix, the acceptance criteria for the bias and RSD for the repeatability and the intermediate precision were adjusted by 5% to ±20% (bias) and ≤ 20% (RSD), respectively. As shown in Table S4, the criteria were fulfilled by all analytes at all three QC levels. The method showed good extraction recovery (exceeding 70%) for all analytes at both low and high concentrations. Significant ion suppression (matrix effects < 75%) was observed for 4‐ANPP, alfentanil, β‐hydroxyfentanyl, fentanyl, hydromorphone, methadone, morphine, norsufentanil, pethidine, sufentanil, and tramadol, and ion enhancement (matrix effects > 125%) was observed for remifentanil acid. As depicted in Table S5, the standard deviations for these analytes remained within acceptable limits (≤ 25%), indicating good reproducibility. The measurements were therefore considered analytically acceptable. Furthermore, the mean relative peak areas (%) of all analytes at the three QC levels, measured in duplicates, remained within ±10% of the reference values after a single freeze–thaw cycle (refer to Table S6). Consequently, stability concerns related to the initial freezing of the skin swab samples during the sampling process are considered negligible.

### 3.2 Participant Characteristics and Medication History

A total of 170 eligible patients were approached for the study, of whom 150 patients were enrolled. The final cohort comprised 59% male (*n* = 88) and 41% female (*n* = 62) participants, with a median age of 53 days (range: 3–5039 days). Body weights ranged from 1.50 to 50.0 kg, with a median of 3.65 kg. A subset of patients underwent organ support therapies during their intensive care treatment, specifically renal replacement therapy (RRT, *n* = 42) or extracorporeal membrane oxygenation (ECMO, *n* = 20). These interventions involved the external connection of the patient's circulatory system to specialized devices to provide blood purification (RRT) and facilitate oxygenation and circulatory support (ECMO). Hair colors ranged from light blonde to black, with light brown being the most common. In most cases, the medication regimen included multiple opioids. As shown in Table 1, the three most frequently administered opioids were fentanyl (*n* = 136), sufentanil (*n* = 108), and morphine (*n* = 141).

**TABLE 1:** Administered opioids and cumulative doses stated in micrograms per kilogram body weight [μg/kgBW]. The values refer to the complete study cohort of 150 patients.

Opioids	*n* cases involving treatment	Cumulative medication dose [μg/kgBW]		
		Median	Mean	Range
Fentanyl	136	19.0	156	0.80–1640
Sufentanil	108	14.0	20.0	0.17–150
Remifentanil	23	34.0	118	5.90–118
Alfentanil	2	32.0	32.0	9.00–54.0
Morphine	141	3130	6510	18.0–90000
Hydromorphone	4	2410	4070	1400–10060
Methadone	18	167	972	64.0–10600

As the median values show, there are considerable variations in the cumulative administered doses between opioids. This variability reflects differences in the dosages and treatment duration and is primarily driven by the respective therapeutic efficacy of the opioids as well as the clinically assessed individual need for pain relief. Details on these characteristics were previously described [1]. For interpretation of the results below, it is important to note that hydromorphone is both a metabolite of morphine and a drug administered independently for analgesia in this study.

### 3.3 Authentic Hair Samples

The analysis of an additional 32 hair samples complemented the findings from the initial 118 hair samples [1] and is summarized in Tables 2 and 3 below.

**TABLE 2:** Positivity rates and obtained analyte concentrations from the authentic hair samples. For an analyte to be counted as detected, the obtained concentration value needed to exceed the respective LOD. Displayed concentration data of the analytes is exclusively based on concentration values [c] above the LLOQ and within the calibration range. Hydromorphone is listed both as therapeutic opioid and as metabolite from morphine (marked with an asterisk). N.A. = not applicable.

Opioids	Positivity rate in hair	*n* cases c > LLOQ	Hair concentration [pg/mg]		
			Median	Mean	Range
Fentanyl	134/136 (99%)	128	4.03	16.1	0.10–343
Sufentanil	76/108 (70%)	68	0.42	0.83	0.10–8.64
Remifentanil	5/23 (22%)	5	0.15	0.20	0.10–0.43
Alfentanil	2/2 (100%)	2	0.21	0.21	0.19–0.22
Morphine	138/141 (98%)	135	31.9	140	1.00–1873
Hydromorphone	4/4 (100%)	4	4.33	6.47	0.90–16.4
Methadone	17/18 (94%)	17	46.1	568	6.00–4990
**Metabolites**					
Norfentanyl	52/136 (38%)	48	1.17	4.43	0.16–123
β‐hydroxyfentanyl	66/136 (49%)	64	1.21	15.1	0.11–559
4‐ANPP	24/136 (18%)	12	0.47	0.82	0.12–2.25
Norsufentanil	37/108 (34%)	34	0.28	0.60	0.10–4.27
Remifentanil acid	0/23 (0%)	0	N.A.	N.A.	N.A.
Hydromorphone*	114/141 (81%)	114	3.97	18.4	0.38–244

**TABLE 3:** Metabolite‐to‐parent drug ratios of the opioids fentanyl, sufentanil, and morphine, obtained from the authentic hair samples.

Metabolites/parent drug	Ratio		
	Median	Mean	Range
Norfentanyl/fentanyl	0.11	0.18	0.02–0.93
β‐hydroxyfentanyl/fentanyl	0.11	0.14	0.01–0.42
4‐ANPP/fentanyl	0.02	0.14	0.0003–0.61
Norsufentanil/sufentanil	0.46	1.06	0.10–6.95
Hydromorphone/morphine	0.10	0.10	0.05–0.23

The evaluation of the complete dataset, comprising 150 hair samples, largely confirmed the preliminarily found positivity rates, concentration ranges, dose–concentration relationships, and metabolite‐to‐parent drug ratios in hair [1]. This was expected, as cumulative opioid doses in the additional 32 cases were within a similar range to those administered in the cases of the preliminary cohort. Findings of particular relevance can be summarized as follows: All administered opioids and their targeted metabolites, with the exception of remifentanil acid, were detected in the hair samples. The opioids most frequently used for medication also exhibited the highest number of detections, with fentanyl detected in 134 of 136 cases (99%), sufentanil in 76 of 108 cases (70%), and morphine in 138 of 141 cases (98%). Hair concentrations of fentanyl, morphine, and methadone were markedly lower than those commonly reported in adult forensic cases [1], whereas sufentanil levels were comparable. Notably, remifentanil was quantified in one additional case, thereby increasing the preliminary dataset to five cases. Concentration ranges of this analyte have never been evaluated in either adult or pediatric hair so far. Statistically significant, moderate positive dose–concentration relationships were observed for fentanyl (*r* = 0.564, *p* < 0.0001) and morphine (*r* = 0.422, *p* < 0.0001), with correlation strengths exceeding those obtained in the preliminary dataset [1]. For sufentanil (*r* = 0.181, *p* = 0.1400) and methadone (*r* = 0.399, *p* = 0.1130), positive yet non‐significant trends were observed, which were consistent with the preliminary results [1].

### 3.4 Authentic Skin Swab Samples

All administered opioids as well as three targeted metabolites were repeatedly detected in the authentic skin swab samples. Table 4 provides the positivity rates and the obtained absolute amounts of analytes per swab (pg/swab). Below, these values will be described in more detail, and resulting metabolite‐to‐parent drug ratios and dose–concentration relationships will be discussed.

**TABLE 4:** Positivity rates and obtained analyte concentrations from the authentic skin swab samples. For an analyte to be counted as detected, the obtained concentration value needed to exceed the respective LOD. Displayed concentration data of the analytes is exclusively based on concentration values [c] above the respective LLOQ values and within the calibration range. Hydromorphone is listed both as therapeutic opioid and as metabolite from morphine (marked with an asterisk). False positives refer to cases with an analytically unambiguous positive analysis result without documented opioid administration. N.A. = not applicable.

Opioids	Positivity rate	False positive	*n* cases c > LLOQ	Skin swab concentration [pg/swab]		
				Median	Mean	Range
Fentanyl	116/136 (85%)	1	92	16.3	36.0	1.01–443
Sufentanil	50/108 (46%)	0	38	5.06	10.1	2.16–73.9
Remifentanil	4/23 (17%)	0	0	N.A.	N.A.	N.A.
Alfentanil	1/2 (50%)	1	0	N.A.	N.A.	N.A.
Morphine	136/141 (96%)	1	118	50.9	153	11.6–1520
Hydromorphone	4/4 (100%)	0	4	15.5	18.8	5.84–38.4
Methadone	8/18 (44%)	0	7	386	1200	78.4–4310
**Metabolites**						
Norfentanyl	3/136 (2%)	1	1	72.8	72.8	N.A.
β‐hydroxyfentanyl	33/136 (24%)	0	17	5.98	31.4	4.81–281
4‐ANPP	0/136 (0%)	0	0	N.A.	N.A.	N.A.
Norsufentanil	0/108 (0%)	0	0	N.A.	N.A.	N.A.
Remifentanil acid	0/23 (0%)	0	0	N.A.	N.A.	N.A.
Hydromorphone*	123/141 (87%)	0	116	5.86	14.0	2.05–116

#### 3.4.1 Fentanyl

Fentanyl was detected in 116 of the 136 cases with reported medication. Concentrations ranged from 1.01 to 443 pg/swab (median: 16.3 pg/swab) and showed a positive, moderately strong correlation (*r* = 0.407, *p* < 0.0001) to administered fentanyl doses. Considering the variability inherent to the skin swab sampling approach, this finding was regarded as significant. Among the three targeted metabolites, norfentanyl and β‐hydroxyfentanyl were repeatedly detected in the fentanyl‐positive samples and exhibited significantly lower concentrations than the parent drug. In the case of β‐hydroxyfentanyl, these concentrations showed a moderate‐to‐strong (*r* = 0.625, *p* = 0.0086) metabolite‐to‐parent drug correlation, with a resulting median metabolite‐to‐parent ratio of 0.11.

#### 3.4.2 Fentanyl Analogs

Among the other opioids belonging to the fentanyl class, for sufentanil, the highest number of detections (50) was recorded. Compared to fentanyl, the positivity rate (46%) was relatively low. As shown in Table 1, sufentanil was administered at the lowest cumulative doses among all opioids, which resulted in comparatively low skin swab sample concentrations (median: 5.06 pg/swab) often close to the limit of quantification. The comparably lower positivity rate observed for sufentanil is therefore likely attributable to the limited sensitivity of the analytical method. No correlation between the measured sufentanil concentrations and the administered opioid doses was observed (*r* = 0.007, *p* = 0.6694). The targeted sufentanil metabolite (norsufentanil) remained undetectable in the corresponding skin swab samples. Remifentanil and alfentanil were administered in 23 and two cases, respectively. While alfentanil was detectable in one case, four positive cases were reported for remifentanil. In addition to extremely low sample concentrations (all below the LLOQ), the poor detectability of remifentanil presumably reflects its extremely short plasma half‐life of only a few minutes, which is attributed to rapid ester hydrolysis. This same metabolic process may also account for the absent detection of its targeted metabolite (remifentanil acid).

#### 3.4.3 Morphine

Morphine was administered most frequently and at the highest cumulative doses, resulting in 136 reported detections out of 141 total cases with documented morphine administration. The median skin swab concentration (50.9 pg/swab) exceeded the levels measured for the comparatively lower‐dosed opioids fentanyl and sufentanil. In contrast, the correlation between skin swab concentrations and administered doses was less pronounced compared to fentanyl but remained statistically significant (*r* = 0.192, *p* = 0.0375). The morphine metabolite hydromorphone was detected in a total of 123 cases, reaching a comparatively high positivity rate of 87%. A strong (*r* = 0.996, *p* < 0.0001) correlation between metabolite and parent drug concentrations was observed, with a resulting median metabolite‐to‐parent ratio of 0.12. Notably, morphine concentrations in cases with a negative result for hydromorphone were low (below 12 pg/swab). These findings are of particular relevance with regard to possible external morphine contamination (refer to the limitations chapter). The formation of hydromorphone is known to be solely possible during body passage [20]. Inconsistency in the hydromorphone/morphine ratios or, respectively, the presence of high morphine concentrations in the absence of hydromorphone could therefore indicate external morphine contamination. Since this was not the case, instances of relevant external contamination of the participants' skin with morphine were considered unlikely.

#### 3.4.4 Hydromorphone

Hydromorphone was detected and quantified in all four cases where it was administered as a therapeutic. All cases involved concomitant morphine treatment, which limits the ability to distinguish between hydromorphone being excreted following its administration as a therapeutic and hydromorphone being excreted as a result of metabolic conversion of morphine. The measured concentrations (range: 5.84–38.4 pg/swab) were within the range observed in morphine cases, where it was exclusively formed through metabolic conversion. The limited sample size did not allow for the evaluation of a dose–concentration relationship.

#### 3.4.5 Methadone

Methadone was detected in eight out of 18 cases with reported medication. The relatively low positivity rate most likely reflected the comparatively low sensitivity (LOD: 40 pg/swab) of the analytical method for this analyte. In contrast, the median skin swab concentration of 386 pg/swab represented the highest median value among all analytes. Notably, the concentration exceeded that of morphine (median: 50.9 pg/swab) despite substantially higher cumulative morphine doses having been administered. Moreover, a positive trend (*r* = 0.829, *p* = 0.0278) between skin swab concentrations and cumulative methadone doses was observed.

To our knowledge, none of the detected analytes have previously been reported in skin‐derived matrices such as sweat or sebum. The only exception was morphine, which had been reported in the sweat of adults, using a substantially different sampling approach involving sweat patches [21, 22]. Consequently, no direct comparison to literature for opioid concentrations was possible. For drugs of abuse belonging to other substance classes, a limited number of studies, employing a similar sampling and analysis approach as here, were found [18, 23]. In the study of Gentili et al. [18] skin swab samples were collected from the foreheads of drivers suspected of drug use during roadside controls and analyzed for classical drugs of abuse, employing gas chromatography–mass spectrometry (GC–MS). Cocaine, amphetamine–type stimulants, and tetrahydrocannabinol (THC) were frequently detected. Notably, the reported concentrations were approximately 1000 times higher than the opioid concentrations measured in the samples of our cohort. Similar high skin swab concentrations were found by Kidwell et al. [23], who measured cocaine via skin swabs obtained from college students. These comparatively high concentration levels may be attributable to higher dosing, drug tolerance, or external contamination, given the typical routes of administration of these substances (smoke and powder dust formation). However, also physical differences in the metabolism or hair structure of adults compared to children could have played a role.

### 3.5 Detection Window of Opioids in Skin‐Derived Matrices (Sweat/Sebum)

The detection windows, defined as the time intervals following the last documented opioid administration during which the analytes remain detectable in the skin swab samples, were assessed and are summarized in Table 5 below.

**TABLE 5:** Detection windows of the analytes, defined as the time intervals following the last documented opioid administration during which the analytes remain detectable in the skin swab samples. Time intervals assigned the value “0” indicate that the opioid treatment continued until the day of sampling. Hydromorphone is listed both as therapeutic opioid and as metabolite from morphine (marked with an asterisk). Analytes with *n* < 4 were not included in the statistics. N.A. = not applicable.

Opioids	*n* skin swab positive cases	Detection window (days)		
		Mean	Range	90% percentile
Fentanyl	116	3.1	0–38	8.0
Sufentanil	50	3.6	0–16	8.0
Remifentanil	4	1.0	N.A.	N.A.
Alfentanil	1	N.A.	N.A.	N.A.
Morphine	136	2.4	0–25	8.3
Hydromorphone	4	3.0	0–12	12
Methadone	8	4.1	0–9	9.0
**Metabolites**				
Norfentanyl	3	N.A.	N.A.	N.A.
β‐hydroxyfentanyl	33	1.4	0–6	4.6
4‐ANPP	0	N.A.	N.A.	N.A.
Norsufentanil	0	N.A.	N.A.	N.A.
Remifentanil acid	0	N.A.	N.A.	N.A.
Hydromorphone*	123	2.1	0–25	7.0

In many cases, the analytes remained detectable for several days, with maximum detection windows reaching 38 days for fentanyl, 16 days for sufentanil, and 25 days for morphine. For opioids, these detection windows in human skin‐derived matrices, following controlled dosing, have not yet been evaluated. However, comparable detection windows have been reported for other substances. In a study by Cho et al. [24], for example, terbinafin was detected in skin swab samples for up to 35 days after oral administration, using thermal desorption‐electrospray ionization tandem mass spectrometry (TD‐ESI/MS/MS). It was assumed that substances excreted over the skin accumulate in sebum and persist in the uneven microstructure of the skin. In contrast, in the study by Kidwell et al. [23], cocaine, which is much less lipophilic than terbinafin, was only detectable on the skin for a maximum of 3 days. The physicochemical characteristics, especially the lipophilicity of a substance, therefore appeared to play an important role for its persistence on the skin. This could therefore explain the prolonged detection window of the highly lipophilic fentanyl evaluated in our study.

### 3.6 Contribution of Skin‐Derived Matrices to Opioid Incorporation Into Hair

**FIGURE 1:**
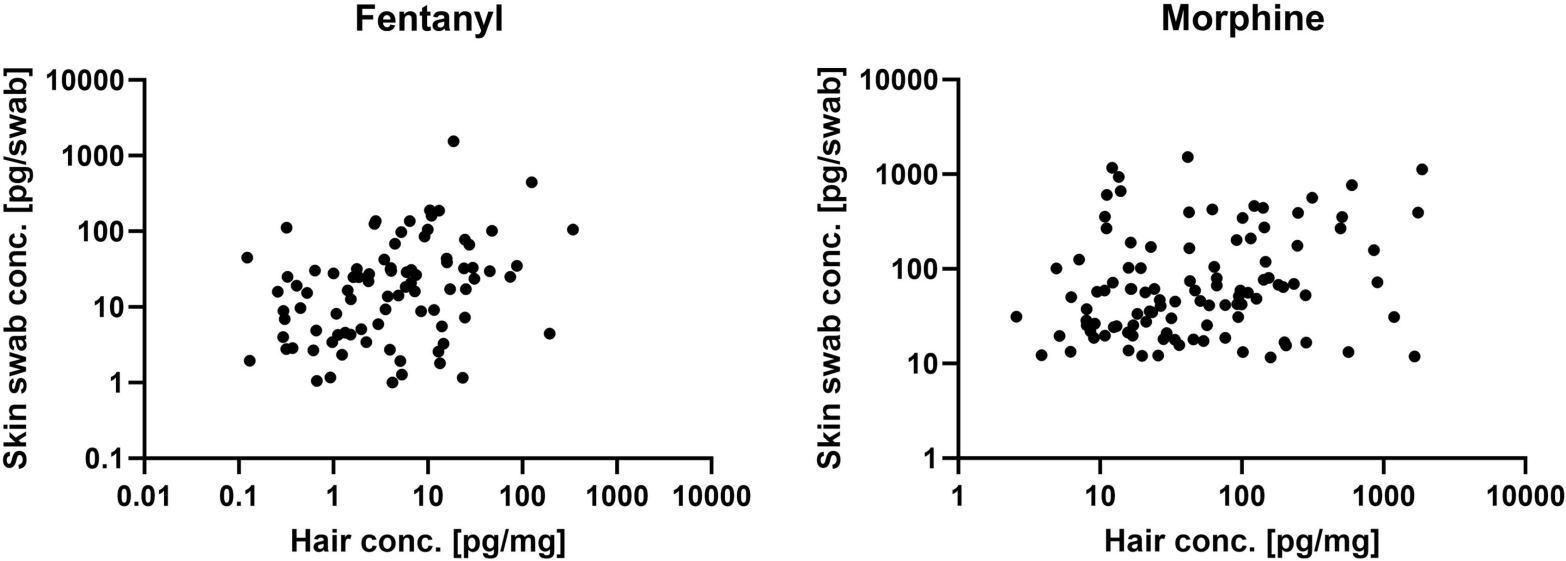
Correlation plots of skin swab and hair sample concentrations for both fentanyl and morphine.

Similar concentration trends and dose–concentration correlations were found in both hair and skin swab samples. Furthermore, the obtained median metabolite‐to‐parent drug ratios in the skin swab samples for β‐hydroxyfentanyl/fentanyl (0.11) and hydromorphone/morphine (0.12) were identical or very similar to those observed in the corresponding hair samples (0.11 and 0.10, respectively). Figure 1 below illustrates skin swab and hair concentration data pairs for fentanyl and morphine, respectively. Based on 87 skin swab and hair concentration data pairs, correlation analysis revealed a statistically significant small‐to‐moderate positive correlation (*r* = 0.356, *p* = 0.0007) for fentanyl. For morphine (105 data pairs), a weaker but still statistically significant correlation was found (*r* = 0.199, *p* = 0.0418).

The similarity of the concentration trends, dose–concentration correlations and metabolite‐to‐parent drug ratios described above supports the hypothesis that skin‐derived matrices may contribute to opioid incorporation into hair via passive diffusion and partitioning processes. However, it must be acknowledged that opioids present systemically in the body are likely a driving force for both follicular (blood‐derived) hair incorporation and analyte concentrations in sweat and sebum. Consequently, the observed correlations between skin swab and hair concentrations for morphine and fentanyl may, at least in part, reflect differences in the administered opioid doses rather than a direct causal contribution of skin‐derived matrices (sweat/sebum) to hair incorporation.

As outlined Section 1, when analytes incorporate into hair via blood circulation through the hair follicle, it requires approximately 7–10 days until they reach the scalp surface. Therefore, this mechanism seems unlikely when opioid administration occurred exclusively within 7 days prior to sampling. Under these conditions, positive analyte findings in hair can most plausibly be explained by incorporation from skin‐derived matrices such as sweat or sebum. In line with this interpretation, high analyte positivity rates were observed in corresponding cases: Fentanyl was detected in 51 of 53 cases, sufentanil in 33 of 45 cases, and morphine in 52 of 55 cases. These findings provide more specific evidence toward the contribution of skin‐derived matrices in the process of opioid incorporation into hair, which seems particularly relevant in the early phase following drug administration.

Notably, sweat and sebum are known to have a distorting influence on dose–concentration relationships in hair. However, the correlation analysis for the skin swab samples suggests that skin‐derived matrices contribute to opioid incorporation into hair in a systematic rather than random manner. This allowed for the presence of pronounced dose–concentration correlations in hair, as they were observed for fentanyl and morphine.

### 3.7 Influence of Covariates on Analyte Concentrations

The influence of patient‐related covariates (age, weight, hair color, sex, and organ support therapies) on analyte concentrations in hair and skin swab samples was evaluated. None of the assessed variables showed an effect on analyte concentrations in skin swab samples. In contrast, age emerged as the only covariate with a significant influence on hair concentrations. Specifically, age was a significant determinant for fentanyl and morphine, with both analytes exhibiting inverse correlations with increasing age (fentanyl: *r* = −0.480, *p* < 0.0001; morphine: *r* = −0.595, *p* < 0.0001). Data from patients below 30 days of age were excluded from the analysis to minimize potential bias related to therapy duration, which in this subgroup was inherently limited by age. Although age‐related variations in dosing could theoretically explain the observed trend, no significant correlation was found between age and cumulative opioid doses normalized to body weight. The elevated hair concentrations observed in younger patients are more plausibly explained by structural characteristics of early‐life hair, particularly its higher porosity, which facilitates radial diffusion and incorporation of substances from sweat and sebum [7].

## 4 Limitations

It is very likely that the skin swab sampling approach applied in this study introduces variability in the collected amounts of skin‐derived matrices, and consequently also in the amounts of analytes obtained. This is probably mainly due to minor differences in parts of the sampling procedure, such as the movement of the swab, the duration of swabbing, and the pressure applied to different anatomical skin areas. In addition, sweat and sebum production are also known to be highly variable, both within and between individuals, depending on factors such as age, anatomical site, temperature, activity, and health status [25]. Furthermore, enzymatic activity on the skin surface, including nonspecific esterases, may further affect the stability and detectability of certain analytes [26]. In addition, hygiene‐related influences such as washing and skin care potentially affect both sweat and sebum composition and the extent of analyte accumulation.

Beyond these physiological and procedural factors, potential external contamination cannot be entirely excluded and could account for the isolated false‐positive results observed in four cases (see Table 4). Drug contamination on clothing or bedding, or contact with medical staff (gloves), could lead to superficial contamination of the participants' skin. According to the study physician at the children's hospital, all opioid administrations within 4 months prior to sampling were documented. This makes an undocumented opioid administration highly unlikely (even though it cannot be completely excluded). Given the small number of affected cases, frequent or significant external contamination is unlikely overall. This interpretation is further supported by the above‐discussed findings made for morphine, in particular the high consistency in the hydromorphone/morphine metabolite‐to‐parent drug ratios.

In summary, the skin swab analysis method is well suited for trend evaluations (detection/non‐detection and relative concentration differences), as performed in this study, but not for absolute quantification. For potential future applications of the method in clinical or forensic contexts, it should further be considered that the study population consisted exclusively of pediatric patients, which limits the extrapolation of the findings to adult populations.

## 5 Conclusion

This clinical study successfully employed validated LC–MS/MS‐based analytical methods to conduct paired analyses of hair and skin swab samples from a pediatric cohort that previously underwent controlled dosing with opioids. We thereby evaluated opioid‐specific concentration ranges, dose–concentration relationships and metabolite‐to‐parent drug ratios in these matrices. The inclusion of detailed patient information and medication records, uniquely available in this study, represents a key strength of this study and confers a high degree of reliability on the data as a reference source. Similar concentration trends and metabolite‐to‐parent drug ratios were found in hair and skin swab samples, with concentrations correlating between the two matrices. Among other findings, this demonstrated the relevance of skin‐derived matrices as a contributor in the process of opioid incorporation into hair. This pathway proved to be particularly relevant in the early stages of hair incorporation and should be considered in cases where opioid exposure exclusively occurred within the first 7 days prior to hair sampling. Notably, the influencing effect of these matrices on dose–concentration correlations appeared to be limited, thereby supporting preliminary findings [1] that hair analysis results in children and neonates can provide valuable supplemental information regarding the underlying extent of opioid intake. Overall, the generated reference data and findings on opioid incorporation pathways addressed a crucial knowledge gap in the literature and will presumably aid future interpretation of hair analysis results in pediatric populations.

## Conflicts of Interest

The authors declare no conflicts of interest.

## Supporting information


**Table S1:** Analytes with their assigned group and internal standard for quantification, calibration ranges, regression type, and obtained limit of detection and lower limit of quantification (LOD and LLOQ). A weighting factor of 1/x was applied to all the calibration models.
**Table S2:** Total analyte concentrations [pg/swab] for the calibrator and QC samples. Each sample was spiked with 50‐μL IS solution (40 pg/μL) resulting in a final swab concentration of 2000 pg/swab for the deuterated analytes.
**Table S3:** Analyte retention times (RT), ion transitions, and optimized compound specific source parameters, including the entrance potential (EP), collision energy (CE), and cell exit potential (CXP) for the positive ionization with electron spray.
**Table S4:** Bias, repeatability and precision values obtained for the quantifier ion of the analytes of the LC–MS/MS method. RSDR = relative standard deviation of repeatability, RSDT = relative standard deviation of time‐different intermediate precision.
**Table S5:** Matrix effects and recoveries in % obtained for the quantifier ion of the analytes of the LC–MS/MS method. Recovery rates were calculated based on normalized signal areas, using the respective internal standards.
**Table S6:** Stability experiment, showing the mean relative peak areas (%) of the analytes, measured in duplicate, compared to a reference after one and three freeze–thaw cycles, respectively.

## Data Availability

The data that support the findings of this study are available on request from the corresponding author. The data are not publicly available due to privacy or ethical restrictions.
