# Supplementary material for: Evaluation of Therapeutic Opioids in Skin‐Derived Matrices (Sweat and Sebum) of Neonatal and Pediatric Patients and Their Role in Opioid Incorporation Into Hair
Source: Drug Test Anal. 2026 Feb 26;18(4):561–71. doi: 10.1002/dta.70034 (PMC13040440; doi:10.1002/dta.70034)
Supplement: Supplementary file 1 — Table S1: Analytes with their assigned group and internal standard for quantification, calibration ranges, regression type, and obtained limit of detection and lower limit of quantification (LOD and LLOQ). A weighting factor of 1/x was applied to all the calibration models. Table S2: Total analyte concentrations [pg/swab] for the calibrator and QC samples. Each sample was spiked with 50‐μL IS solution (40 pg/μL) resulting in a final swab concentration of 2000 pg/swab for the deuterated analytes. Table S3: Analyte retention times (RT), ion transitions, and optimized compound specific source parameters, including the entrance potential (EP), collision energy (CE), and cell exit potential (CXP) for the positive ionization with electron spray. Table S4: Bias, repeatability and precision values obtained for the quantifier ion of the analytes of the LC–MS/MS method. RSDR = relative standard deviation of repeatability, RSDT = relative standard deviation of time‐different intermediate precision. Table S5: Matrix effects and recoveries in % obtained for the quantifier ion of the analytes of the LC–MS/MS method. Recovery rates were calculated based on normalized signal areas, using the respective internal standards. Table S6: Stability experiment, showing the mean relative peak areas (%) of the analytes, measured in duplicate, compared to a reference after one and three freeze–thaw cycles, respectively. [file DTA-18-561-s001.docx]

Table S1: Analytes with their assigned group and internal standard for quantification, calibration ranges, regression type and obtained limit of detection and lower limit of quantification (LOD and LLOQ). A weighting factor of 1/x was applied to all the calibration models.

| **Analyte / Group** | **Internal standard (IS)** | **Calibration range**  **[pg/swab]** | **Regression** | **LOD**  **[pg/swab]** | **LLOQ [pg/swab]** | **Linearity (R^2^)** |
| --- | --- | --- | --- | --- | --- | --- |
| 4-ANPP / 2 | Fentanyl-d_5_ | 2 - 1'600 | linear | 1.5 | 2.0 | 0.9942 |
| Acetylcodeine / 5 | Morphine-d_3_ | 10 - 16'000 | linear | 1.0 | 10 | 0.9977 |
| 6-Monoacetylmorphine / 5 | Morphine-d_3_ | 10 - 16'000 | linear | 1.5 | 10 | 0.9962 |
| Alfentanil / 1 | Fentanyl-d_5_ | 1 - 1'600 | linear | 0.2 | 1.0 | 0.9943 |
| β-Hydroxyfentanyl / 3 | Fentanyl-d_5_ | 4 - 1'600 | linear | 1.5 | 4.0 | 0.9954 |
| Codeine / 5 | Morphine-d_3_ | 10 - 16'000 | linear | 1.5 | 10 | 0.9957 |
| Dihydrocodeine / 5 | Morphine-d_3_ | 10 - 16'000 | linear | 1.0 | 10 | 0.9956 |
| Fentanyl / 1 | Fentanyl-d5 | 1 - 1'600 | linear | 0.4 | 1.0 | 0.9989 |
| Hydrocodone / 2 | Morphine-d_3_ | 2 - 1'600 | linear | 1.0 | 2.0 | 0.9920 |
| Hydromorphone / 2 | Morphine-d_3_ | 2 - 1'600 | linear | 1.5 | 2.0 | 0.9988 |
| Methadone / 6 | Methadone-d_9_ | 60 - 16'000 | linear | 40 | 60 | 0.9991 |
| Morphine / 5 | Morphine-d_3_ | 10 - 16'000 | linear | 3.0 | 10 | 0.9991 |
| Naloxone / 5 | Morphine-d_3_ | 10 - 16'000 | linear | 2.0 | 10 | 0.9984 |
| Norfentanyl / 4 | Norfentanyl-d_5_ | 6 - 1'600 | linear | 2.5 | 6.0 | 0.9990 |
| Norsufentanil / 2 | Norsufentanil-d_3_ | 2 - 1'600 | linear | 1.0 | 2.0 | 0.9989 |
| Oxycodone / 5 | Morphine-d_3_ | 10 - 16'000 | linear | 1.0 | 10 | 0.9955 |
| Oxymorphone / 5 | Morphine-d_3_ | 10 - 16'000 | linear | 1.0 | 10 | 0.9962 |
| Pethidine / 2 | Morphine-d_3_ | 2 - 1'600 | linear | 1.0 | 2.0 | 0.9982 |
| Remifentanil / 2 | Fentanyl-d_5_ | 2 - 1'600 | linear | 1.0 | 2.0 | 0.9955 |
| Remifentanil-acid / 4 | Fentanyl-d_5_ | 6 - 1'600 | linear | 2.0 | 6.0 | 0.9758 |
| Sufentanil / 2 | Fentanyl-d_5_ | 2 - 1'600 | linear | 1.0 | 2.0 | 0.9976 |
| Tramadol / 5 | Methadone-d_9_ | 10 - 16'000 | linear | 4.0 | 10 | 0.9953 |

Table S2: Total analyte concentrations [pg/swab] for the calibrator and QC samples. Each sample was spiked with 50 µL IS solution (40 pg/µL) resulting in a final swab concentration of 2000 pg/swab for the deuterated analytes.

| **Group** | **Analyte concentrations in swab [pg/swab]** | | | | | | | | | | |
| --- | --- | --- | --- | --- | --- | --- | --- | --- | --- | --- | --- |
|  | **Cal. 1** | **Cal. 2** | **Cal. 3** | **Cal. 4** | **Cal. 5** | **Cal. 6** | **Cal. 7** | **Cal. 8** | **QC**_low_ | **QC**_med_ | **QC**_high_ |
| 1 | 1 | 2 | 10 | 20 | 100 | 200 | 1'000 | 1'600 | 1.2 | 180 | 1'400 |
| 2 | 2 | 4 | 10 | 20 | 100 | 200 | 1'000 | 1'600 | 2.4 | 180 | 1'400 |
| 3 | 4 | 8 | 12 | 24 | 100 | 200 | 1'000 | 1'600 | 4.8 | 216 | 1'400 |
| 4 | 6 | 12 | 18 | 36 | 100 | 200 | 1'000 | 1'600 | 7.2 | 324 | 1'400 |
| 5 | 10 | 20 | 100 | 200 | 1'000 | 2'000 | 10'000 | 16'000 | 12 | 1'800 | 14'000 |
| 6 | 60 | 120 | 100 | 200 | 1'000 | 2'000 | 10'000 | 16'000 | 72 | 1'800 | 14'000 |

Table S3: Analyte retention times (RT), ion transitions and optimized compound specific source parameters, including the entrance potential (EP), collision energy (CE) and cell exit potential (CXP) for the positive ionization with electron spray.

| **Substance** | **Q1 mass [Da]** | | **Q3 mass [Da]** | | **RT [min]** | **EP [V]** | **CE [V]** | **CXP [V]** |
| --- | --- | --- | --- | --- | --- | --- | --- | --- |
| 4-ANPP | | 281.2 | | 188.1/105.0 | 5.88 | 10 | 23/39 | 10/12 |
| Acetylcodeine | | 342.1 | | 152.0/165.1 | 4.58 | 10 | 91/59 | 12/12 |
| 6-Monoacetylmorphine | | 328.1 | | 164.9/211.0 | 3.56 | 10 | 51/35 | 14/16 |
| Alfentanil | | 417.2 | | 268.1/197.1 | 5.59 | 10 | 23/25 | 14/10 |
| β-Hydroxyfentanyl | | 353.2 | | 204.0/146.0 | 5.34 | 10 | 30/33 | 13/10 |
| Codeine | | 300.0 | | 215.2/165.1 | 3.12 | 10 | 33/53 | 20/16 |
| Dihydrocodeine | | 302.1 | | 201.2/171.1 | 3.08 | 10 | 37/55 | 18/10 |
| Fentanyl | | 337.2 | | 188.1/105.0 | 5.84 | 10 | 32/53 | 13/12 |
| Hydrocodone | | 300.1 | | 199.1/128.1 | 3.66 | 10 | 41/75 | 16/12 |
| Hydromorphone | | 286.1 | | 157.1/185.0 | 1.22 | 10 | 55/39 | 14/16 |
| Methadone | | 310.2 | | 91.0/219.0 | 6.77 | 10 | 72/31 | 12/18 |
| Morphine | | 286.1 | | 152.1/128.1 | 1.23 | 10 | 83/77 | 18/14 |
| Naloxone | | 328.1 | | 212.1/310.2 | 3.18 | 10 | 51/27 | 24/20 |
| Norfentanyl | | 233.1 | | 150.0/177.1 | 3.94 | 10 | 18/21 | 10/22 |
| Norsufentanil | | 277.2 | | 128.0/245.2 | 4.74 | 10 | 19/27 | 6/12 |
| Oxycodone | | 316.1 | | 212.2/187.1 | 3.45 | 10 | 55/35 | 18/14 |
| Oxymorphone | | 302.0 | | 198.0/284.1 | 1.56 | 10 | 59/27 | 16/18 |
| Pethidine | | 248.1 | | 220.1/174.1 | 4.78 | 10 | 29/29 | 20/10 |
| Remifentanil | | 377.1 | | 228.1/317.0 | 4.90 | 10 | 27/21 | 12/16 |
| Remifentanil-acid | | 363.3 | | 113.1/303.0 | 4.46 | 10 | 30/21 | 18/18 |
| Sufentanil | | 387.1 | | 238.0/355.0 | 6.53 | 10 | 27/25 | 12/18 |
| Tramadol | | 264.1 | | 42.0/58.0 | 4.45 | 10 | 103/49 | 6/10 |
| Morphine-d_3_ | | 289.2 | | 201.0 | 1.23 | 10 | 37 | 13 |
| Norsufentanil-d_3_ | | 280.1 | | 131.0 | 4.73 | 10 | 19 | 8 |
| Fentanyl-d_5_ | | 342.2 | | 137.2 | 5.82 | 10 | 45 | 8 |
| Norfentanyl-d_5_ | | 238.2 | | 182.1 | 3.94 | 10 | 23 | 12 |
| Methadone-d_9_ | | 319.3 | | 268.1 | 6.77 | 10 | 23 | 16 |

Table S4: Bias, repeatability and precision values obtained for the quantifier ion of the analytes of the LC–MS/MS method. RSDR = relative standard deviation of repeatability, RSDT = relative standard deviation of time-different intermediate precision.

| **Substance** | **QC Level** | **Theoretical conc.**  **[pg/swab]** | **Calculated conc.**  **[pg/swab]** | **Bias [%]** | **RSD_R_ [%]** | **RSD_T_ [%]** |
| --- | --- | --- | --- | --- | --- | --- |
| 4-ANPP | low | 2.4 | 2.50 | 3.50 | 2.80 | 5.50 |
|  | med | 180 | 177 | -1.40 | 6.20 | 8.00 |
|  | high | 1'400 | 1'415 | 1.10 | 13.9 | 11.6 |
| Acetylcodeine | low | 12 | 12.6 | 4.60 | 3.10 | 7.40 |
|  | med | 1'800 | 1'736 | -3.60 | 7.70 | 6.80 |
|  | high | 14'000 | 13'006 | -7.10 | 7.40 | 9.00 |
| 6-Monoacetylmorphine | low | 12 | 11.70 | -2.10 | 9.20 | 8.20 |
|  | med | 1'800 | 1'740 | -3.30 | 6.60 | 7.60 |
|  | high | 14'000 | 12'862 | -8.10 | 6.70 | 9.60 |
| Alfentanil | low | 1.2 | 1.20 | 3.50 | 3.50 | 9.30 |
|  | med | 180 | 164 | -8.70 | 6.60 | 6.60 |
|  | high | 1'400 | 1'254 | -10.4 | 8.80 | 8.60 |
| β-Hydroxyfentanyl | low | 4.8 | 4.80 | -0.70 | 1.90 | 2.40 |
|  | med | 216 | 199 | -7.90 | 6.40 | 5.30 |
|  | high | 1'400 | 1'269 | -9.40 | 7.40 | 6.10 |
| Codeine | low | 12 | 11.4 | -5.10 | 3.50 | 9.50 |
|  | med | 1'800 | 1'868 | 3.80 | 3.50 | 5.30 |
|  | high | 14'000 | 13'550 | -3.20 | 5.30 | 11.7 |
| Dihydrocodeine | low | 12 | 11.0 | -8.30 | 9.20 | 10.0 |
|  | med | 1'800 | 1'880 | 4.40 | 3.50 | 5.60 |
|  | high | 14'000 | 13'497 | -3.60 | 3.30 | 11.2 |
| Fentanyl | low | 1.2 | 1.20 | 1.20 | 3.20 | 2.80 |
|  | med | 180 | 175 | -3.10 | 3.00 | 3.80 |
|  | high | 1'400 | 1'323 | -5.50 | 2.80 | 3.50 |
| Hydrocodone | low | 2.4 | 2.20 | -6.90 | 2.00 | 7.70 |
|  | med | 180 | 171 | -5.00 | 5.60 | 7.60 |
|  | high | 1'400 | 1'384 | -1.10 | 8.50 | 9.80 |
| Hydromorphone | low | 2.4 | 2.30 | -2.30 | 5.60 | 6.70 |
|  | med | 180 | 179 | -0.40 | 1.50 | 3.00 |
|  | high | 1'400 | 1'374 | -1.90 | 4.70 | 6.20 |
| Methadone | low | 72 | 71.5 | -0.60 | 1.60 | 3.10 |
|  | med | 1'800 | 1'823 | 1.30 | 1.00 | 4.70 |
|  | high | 14'000 | 13'322 | -16.7 | 1.40 | 2.60 |
| Morphine | low | 12 | 11.8 | -2.00 | 1.20 | 2.10 |
|  | med | 1'800 | 1'765 | -1.90 | 1.60 | 4.20 |
|  | high | 14'000 | 13'557 | -3.20 | 3.50 | 5.40 |
| Naloxone | low | 12 | 11.9 | -1.20 | 2.40 | 3.20 |
|  | med | 1'800 | 1'850 | 2.80 | 2.60 | 3.00 |
|  | high | 14'000 | 13'882 | -0.80 | 4.00 | 5.10 |
| Norfentanyl | low | 7.2 | 7.30 | 1.80 | 1.90 | 2.00 |
|  | med | 324 | 308 | -5.00 | 1.90 | 4.20 |
|  | high | 1'400 | 1'346 | -3.90 | 2.90 | 4.70 |
| Norsufentanil | low | 2.4 | 2.40 | 1.20 | 1.80 | 3.40 |
|  | med | 180 | 169 | -6.10 | 2.70 | 2.60 |
|  | high | 1'400 | 1'319 | -5.80 | 3.60 | 3.00 |
| Oxycodone | low | 12 | 12.2 | 1.80 | 9.10 | 7.20 |
|  | med | 1'800 | 1'869 | 3.80 | 3.80 | 7.70 |
|  | high | 14'000 | 13'593 | -2.90 | 5.10 | 12.0 |
| Oxymorphone | low | 12 | 11.8 | -1.60 | 9.60 | 7.60 |
|  | med | 1'800 | 1'856 | 3.10 | 4.00 | 6.40 |
|  | high | 14'000 | 13'440 | -4.00 | 4.00 | 10.3 |
| Pethidine | low | 2.4 | 2.40 | 0.70 | 2.70 | 8.60 |
|  | med | 180 | 168 | -6.60 | 5.90 | 4.80 |
|  | high | 1'400 | 1'301 | -7.10 | 4.60 | 8.30 |
| Remifentanil | low | 2.4 | 2.40 | 0.50 | 1.70 | 10.7 |
|  | med | 180 | 160 | -11.2 | 5.20 | 10.6 |
|  | high | 1'400 | 1'175 | -16.1 | 9.10 | 17.0 |
| Remifentanil-acid | low | 7.2 | 7.00 | -2.80 | 3.80 | 4.30 |
|  | med | 324 | 341 | 5.10 | 10.7 | 13.4 |
|  | high | 1'400 | 1'442 | 3.00 | 16.6 | 18.2 |
| Sufentanil | low | 2.4 | 2.50 | 4.50 | 3.30 | 4.90 |
|  | med | 180 | 172 | -4.30 | 2.50 | 6.30 |
|  | high | 1'400 | 1'374 | -1.90 | 8.90 | 8.20 |
| Tramadol | low | 12 | 11.9 | -0.80 | 2.20 | 8.20 |
|  | med | 1'800 | 1'803 | 0.20 | 4.90 | 4.70 |
|  | high | 14'000 | 13'038 | -6.90 | 10.9 | 11.5 |

Table S5: Matrix effects and recoveries in % obtained for the quantifier ion of the analytes of the LC–MS/MS method. Recovery rates were calculated based on normalized signal areas, using the respective internal standards.

| **Substance** | **QC Sample** | **Theoretical conc.**  **[pg/swab]** | **Recovery** | | **Matrix effect** | |
| --- | --- | --- | --- | --- | --- | --- |
|  |  |  | **Mean [%]** | **SD [%]** | **Mean [%]** | **SD [%]** |
| 4-ANPP | low | 2.4 | 85.9 | 16.2 | 34.2 | 24.5 |
|  | high | 1'400 | 95.5 | 34.8 | 29.8 | 16.7 |
| Acetylcodeine | low | 12 | 97.6 | 14.9 | 91.9 | 16.6 |
|  | high | 14'000 | 86.3 | 9.00 | 88.8 | 7.10 |
| 6-Monoacetylmorphine | low | 12 | 104 | 7.20 | 95.4 | 18.8 |
|  | high | 14'000 | 92.7 | 10.8 | 76.1 | 12.9 |
| Alfentanil | low | 1.2 | 91.2 | 24.3 | 75.3 | 22.9 |
|  | high | 1'400 | 85.2 | 22.0 | 52.7 | 9.70 |
| β-Hydroxyfentanyl | low | 4.8 | 85.7 | 24.2 | 68.5 | 21.9 |
|  | high | 1'400 | 85.4 | 24.1 | 52.5 | 8.90 |
| Codeine | low | 12 | 104 | 19.5 | 99.6 | 10.0 |
|  | high | 14'000 | 94.4 | 2.50 | 95.2 | 7.20 |
| Dihydrocodeine | low | 12 | 97.7 | 5.30 | 89.8 | 11.6 |
|  | high | 14'000 | 88.7 | 2.60 | 89.8 | 6.50 |
| Fentanyl | low | 1.2 | 106 | 33.5 | 45.3 | 20.7 |
|  | high | 1'400 | 94.4 | 33.8 | 40.8 | 8.30 |
| Hydrocodone | low | 2.4 | 104 | 17.3 | 121 | 12.9 |
|  | high | 1'400 | 90.8 | 13.7 | 98.2 | 13.1 |
| Hydromorphone | low | 2.4 | 95.2 | 13.9 | 78.9 | 16.0 |
|  | high | 1'400 | 85.1 | 13.0 | 47.5 | 9.90 |
| Methadone | low | 72 | 112 | 22.5 | 57.2 | 19.4 |
|  | high | 14'000 | 106 | 23.0 | 56.7 | 8.20 |
| Morphine | low | 12 | 94.7 | 15.9 | 75.7 | 12.4 |
|  | high | 14'000 | 96.5 | 7.00 | 50.3 | 8.00 |
| Naloxone | low | 12 | 98.4 | 13.1 | 112 | 12.2 |
|  | high | 14'000 | 92.6 | 13.1 | 86.2 | 6.90 |
| Norfentanyl | low | 7.2 | 85.2 | 8.60 | 102 | 9.70 |
|  | high | 1'400 | 87.5 | 6.70 | 102 | 8.70 |
| Norsufentanil | low | 2.4 | 88.9 | 22.5 | 86.8 | 14.7 |
|  | high | 1'400 | 79.9 | 7.60 | 74.0 | 6.50 |
| Oxycodone | low | 12 | 104 | 8.40 | 123 | 14.7 |
|  | high | 14'000 | 98.1 | 6.70 | 93.8 | 14.6 |
| Oxymorphone | low | 12 | 95.2 | 3.70 | 102 | 9.10 |
|  | high | 14'000 | 90.0 | 3.10 | 78.5 | 17.9 |
| Pethidine | low | 2.4 | 87.4 | 12.2 | 35.9 | 17.2 |
|  | high | 1'400 | 91.8 | 19.3 | 39.0 | 10.2 |
| Remifentanil | low | 2.4 | 103 | 16.3 | 102 | 14.2 |
|  | high | 1'400 | 87.7 | 14.1 | 82.0 | 7.10 |
| Remifentanil-acid | low | 7.2 | 98.5 | 8.00 | 237 | 9.50 |
|  | high | 1'400 | 94.9 | 20.7 | 176 | 10.6 |
| Sufentanil | low | 2.4 | 109 | 24.8 | 36.3 | 21.7 |
|  | high | 1'400 | 104 | 39.2 | 32.2 | 14.4 |
| Tramadol | low | 12 | 98.0 | 19.6 | 83.5 | 13.8 |
|  | high | 14'000 | 86.4 | 9.30 | 71.3 | 11.2 |

Table S6: Stability experiment, showing the mean relative peak areas (%) of the analytes, measured in duplicate, compared to a reference after one and three freeze–thaw cycles, respectively.

| **Substance** | **QC Level** | **One cycle**  **Relative peak area [%]** | **Three cycles**  **Relative peak area [%]** |
| --- | --- | --- | --- |
| 4-ANPP | low | 103 | 77.3 |
|  | med | 96.4 | 84.9 |
|  | high | 97.8 | 81.0 |
| Acetylcodeine | low | 94.0 | 79.7 |
|  | med | 96.9 | 72.3 |
|  | high | 98.1 | 70.6 |
| 6-Monoacetylmorphine | low | 101 | 47.4 |
|  | med | 95.6 | 42.3 |
|  | high | 97.9 | 43.0 |
| Alfentanil | low | 99.8 | 76.2 |
|  | med | 100 | 73.8 |
|  | high | 101 | 77.5 |
| β-Hydroxyfentanyl | low | 103 | 68.5 |
|  | med | 96.4 | 74.4 |
|  | high | 99.3 | 79.0 |
| Codeine | low | 94.2 | 80.3 |
|  | med | 103 | 74.5 |
|  | high | 103 | 79.7 |
| Dihydrocodeine | low | 96.4 | 83.5 |
|  | med | 97.7 | 73.8 |
|  | high | 97.6 | 77.0 |
| Fentanyl | low | 97.9 | 75.6 |
|  | med | 98.2 | 76.6 |
|  | high | 99.3 | 79.1 |
| Hydrocodone | low | 105 | 43.6 |
|  | med | 104 | 55.4 |
|  | high | 107 | 56.3 |
| Hydromorphone | low | 90.4 | 68.6 |
|  | med | 106 | 54.6 |
|  | high | 98.6 | 62.8 |
| Methadone | low | 94.0 | 77.7 |
|  | med | 98.0 | 73.9 |
|  | high | 98.9 | 71.4 |
| Morphine | low | 97.7 | 63.1 |
|  | med | 104 | 53.4 |
|  | high | 98.3 | 59.8 |
| Naloxone | low | 98.4 | 72.0 |
|  | med | 103 | 77.1 |
|  | high | 99.6 | 73.9 |
| Norfentanyl | low | 94.3 | 73.6 |
|  | med | 96.9 | 65.6 |
|  | high | 97.9 | 68.3 |
| Norsufentanil | low | 94.1 | 77.2 |
|  | med | 103 | 78.2 |
|  | high | 97.3 | 78.2 |
| Oxycodone | low | 98.5 | 72.3 |
|  | med | 95.7 | 65.2 |
|  | high | 102 | 70.3 |
| Oxymorphone | low | 97.8 | 65.6 |
|  | med | 100 | 71.4 |
|  | high | 101 | 74.4 |
| Pethidine | low | 101 | 81.7 |
|  | med | 97.8 | 77.3 |
|  | high | 99.9 | 74.5 |
| Remifentanil | low | 101 | 37.1 |
|  | med | 69.4 | 40.9 |
|  | high | 97.8 | 39.4 |
| Remifentanil-acid | low | 108 | 84.2 |
|  | med | 106 | 96.2 |
|  | high | 110 | 96.8 |
| Sufentanil | low | 98.3 | 63.9 |
|  | med | 100 | 68.1 |
|  | high | 98.2 | 69.1 |
| Tramadol | low | 103 | 69.0 |
|  | med | 98.2 | 79.1 |
|  | high | 100 | 78.1 |
